# Surgical versus interventional coronary revascularization in kidney transplant recipients: a systematic review and meta-analysis

**DOI:** 10.1007/s11255-023-03546-9

**Published:** 2023-03-12

**Authors:** Amr Ehab El-Qushayri, Abdullah Reda

**Affiliations:** 1 Faculty of Medicine, Minia University, Minia, Egypt; 2 Faculty of Medicine, Al-Azhar University, Cairo, Egypt

**Keywords:** PCI, CABG, Renal transplantation, Systematic review, Meta-analysis

## Abstract

**Aim:**

To study the most beneficial coronary revascularization strategy in kidney transplant recipients (KTR).

**Methods:**

In 16th June 2022 and updated on 26th February 2023, we searched in five databases including PubMed for relevant articles. The odds ratio (OR) together with the 95% confidence interval (95%CI) were used to report the results.

**Results:**

Percutaneous coronary intervention (PCI) was significantly associated with significant lower in-hospital mortality (OR 0.62; 95%CI 0.51–0.75) and 1-year mortality (OR 0.81; 95%CI 0.68–0.97), but not overall mortality (mortality at the last follow-up point) (OR 1.05; 95%CI 0.93–1.18) rather than coronary artery bypass graft (CABG). Moreover, PCI was significantly associated with lower acute kidney injury prevalence (OR 0.33; 95%CI 0.13–0.84) compared to CABG. One study indicated that non-fatal graft failure prevalence did not differ between the PCI and the CABG group until 3 years of follow up. Moreover, one study demonstrated a short hospital length of stay in the PCI group rather than the CABG group.

**Conclusion:**

Current evidence indicated the superiority of PCI than CABG as a coronary revascularization procedure in short- but not long-term outcomes in KTR. We recommend further randomized clinical trials for demonstrating the best therapeutic modality for coronary revascularization in KTR.

**Supplementary Information:**

The online version contains supplementary material available at 10.1007/s11255-023-03546-9.

## Introduction

In the recent years, a rising incidence of end stage renal disease (ESRD) was observed [1]. Only few treatment options are available for ESRD patients including hemodialysis and kidney transplantation (KT). Cumulative evidence from a systematic review indicated the superiority of KT over chronic hemodialysis regarding short- and long-term clinical outcomes. Moreover, the study demonstrated a beneficial effect of KT in reducing the rates of all cardiovascular events rather than dialysis option [2].

Estimates showed a high risk of cardiac and all-cause mortality among patients with chronic kidney disease (CKD) [3]. Moreover, cardiovascular disease and related mortality risks are high among kidney transplant recipients (KTRs) [4]. Revascularization strategies are valuable options for these patients. However, previous studies have focused on CKD and ESRD patients [5, 6]. On the other hand, only some studies have been conducted on KTRs, with no cumulative evidence regarding the most effective revascularization strategy [7–10]. In a population based study of Charytan et al. for KTRs, Coronary Artery Bypass Graft Surgery (CABG) patients had a significantly higher mortality rate rather than patients who received Percutaneous Coronary Intervention (PCI) procedure after 3 months of follow up, and the significance was lost till the end of the follow up period (3 years) [7]. Furthermore, Lang et al. demonstrated no differences in the in-hospital mortality rates or after four years of follow up between the CABG and the PCI groups [8]. The same observation was also noticed by Taduru et al. where there was no difference regarding the in-hospital mortality rates between the two revascularization techniques [9]. The superiority of each technique should not only be based upon health outcomes, but also economic costs as well. Due to such variability in the beneficial effect of one technique over another, we aimed to conduct this meta-analysis to investigate which modality is better regarding the clinical outcomes in KTRs; CABG or PCI.

## Methods

### Study selection

In 16th June 2022 and updated on 26th February 2023, a literature search that followed the PRISMA guidelines was conducted in five databases (Google Scholar, Scopus, Web of Science, PubMed and Virtual Health Library) using the search term “("renal transplantation" OR "renal transplant") AND ("percutaneous coronary intervention" OR PCI OR "coronary catheterization" OR "coronary stenting") AND ("coronary artery bypass graft" OR "CABG") (Table S1). Two authors did the screening (title and abstract then full text screening) and the extraction processes of the resulted records according to the eligibility criteria: any study reported the comparison between PCI and CABG as coronary revascularization procedure in KTRs were included without applying any restrictions to age, sex, race and other comorbid conditions. While we excluded conference abstracts, duplicate studies with the same patients and studies with only one arm of coronary revascularization procedure, review papers and not relevant studies.

We extracted all the characteristic information from all studies including: male prevalence, study design, study ID, age, sample size and comorbidities. Moreover, our outcomes consisted of length of hospital stay, mortality, graft failure and acute kidney injury (AKI). In both steps of the screening and the extraction, a discussion was started if disagreement occurred to ensure a clean data.

### Quality assessment

We used the well-known quality assessment tool of The National Institute of Health for observational studies [11]. The tool divided the quality of studies into three types, good, fair and poor quality (Table S2).

### Statistical analysis

Comprehensive meta-analysis software was used to analyze the results. We used the events of each outcome and the total sample size from each study to calculate the odds ratio (OR) and the 95% confidence interval (95%CI) as the pooled estimate from all the included papers in our meta-analysis. Random effect model was chosen if *p* value of heterogeneity was less than 0.1 or I2 more than 50. The significance of the results was obtained when *p* value falls below 0.05. Publication bias and meta-regression analyses were not applicable in our study as the needed number of the included studies should be ≥ 10 [12].

## Results

### Study results and characteristics

**Fig. 1:**
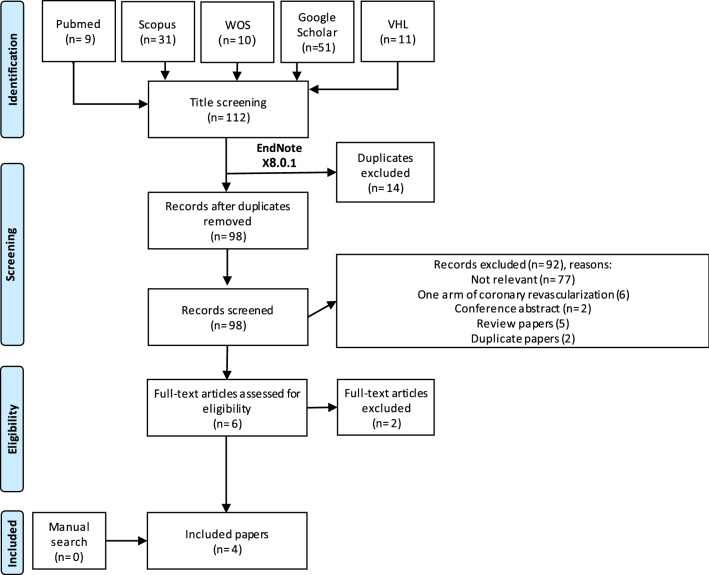
PRIMSA flow diagram of the study process

Of total 98 records screened, we included 4 retrospective cohort studies, 6674 and 4402 KTRs underwent for PCI and CABG, respectively (Fig. 1 and Table 1) [7–10]. Three studies were conducted in USA and one in Germany. All studies obtained fair criterion according to the National Institute of Health quality assessment tool (Table S2).

**Table 1:** Characteristics of the included papers

Study ID	Study design	Compared groups	Sample size	Age	Male %	CHF %	Arrhythmia %	MI %	Cancer %	DM	Hypertension	Follow up
Charytan-2015-USA	Retrospective cohort	PCI/CABG	4097/1400	> 45*#	66/69	45/51	40/50	51/47	7/6	65/69	–	3 years
Lang-2018-Germany	Retrospective cohort	PCI/CABG	27/24	64/62**	78/54	–	–	–	–	33/25	100/100	4 years
Taduru-2017-USA	Retrospective cohort	PCI/CABG	1871/1878	61/61***	66/69	24/23	36/37	–	–	59/60	79/68	–
Herzog-2004-USA	Retrospective cohort	PCI/CABG	652/1100	> 45*#	68/71	–	–	–	–	–	–	32 months for PCI 25.4 months for CABG

*CHF* congestive heart failure, *MI* myocardial infarction, *DM* diabetes mellitus

*Range, **median,***mean, #both groups

### Mortality

**Fig. 2:**
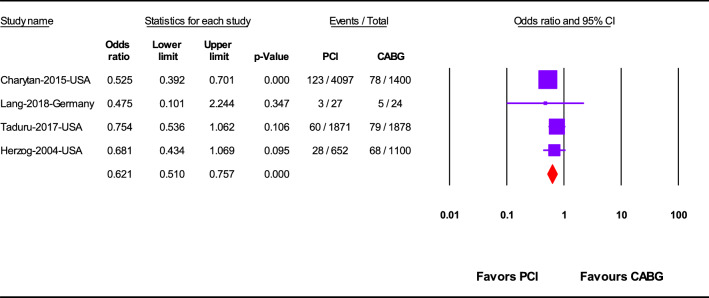
In-hospital mortality after revascularization by PCI or CABG

**Fig. 3:**
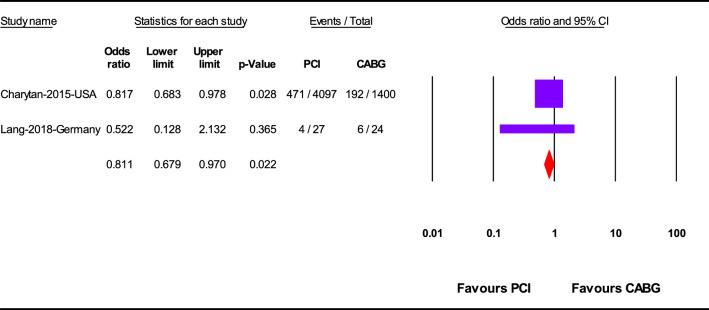
1-Year mortality after revascularization by PCI or CABG

**Fig. 4:**
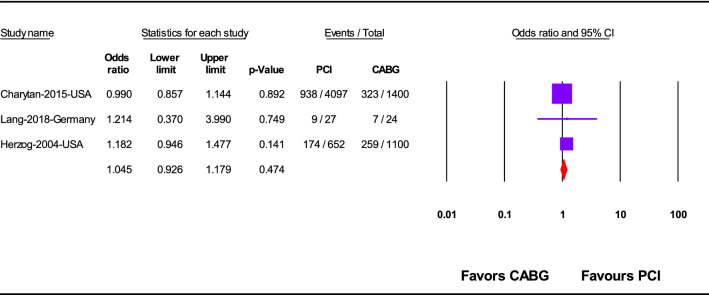
Overall mortality after revascularization by PCI or CABG

PCI was significantly associated with significant lower in-hospital mortality (OR 0.62; 95%CI 0.51–0.75; *p* < 0.001) (Fig. 2) and 1-year mortality (OR 0.81; 95%CI 0.68–0.97; *p* = 0.02) (Fig. 3), but not overall mortality (mortality at the last follow up point) (OR 1.05; 95%CI 0.93–1.18; *p* = 0.47) rather than CABG (Fig. 4).

### AKI

**Fig. 5:**
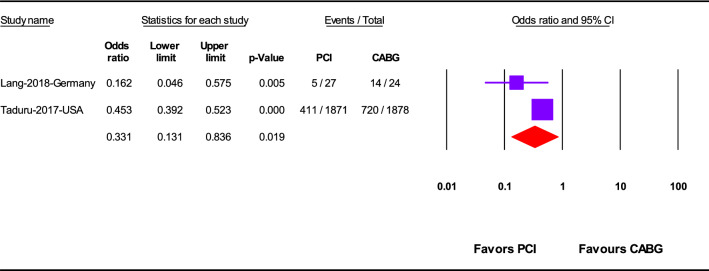
Acute kidney injury after revascularization by PCI or CABG

PCI was significantly associated with lower AKI prevalence (OR 0.33; 95%CI 0.13–0.84; *p* = 0.02) compared to CABG (Fig. 5).

### Graft failure

One study indicated that non-fatal graft failure prevalence did not differ between the PCI and the CABG group until 3 years of follow-up [7].

### Length of hospital stay

One study demonstrated shorted hospital length of stay in the PCI group rather than the CABG group (*p* < 0.001) [9].

## Discussion

Our findings showed that PCI induced better outcomes than CABG in KTRs, regarding in-hospital, 1-year mortality and AKI prevalence rates. These findings are consistent with the previous investigations that demonstrated that PCI is more effective than CABG in reducing mortality rates among the different patient groups, including cardiomyopathy and heart transplant recipients with coronary allograft vasculopathy [13, 14].

We furtherly found that the prevalence of non-graft failure did not differ between the two groups. However, such evidence was obtained from a single investigation indicating the non-significant short and long-term difference between the two modalities. Moreover, Bagheri et al. [14] showed that the 5-year survival outcomes favored PCI over CABG in cardiomyopathy patients. However, we did not find a significant difference at the last follow-up point, regarding all-cause mortality, which is also consistent with previous evidence [15]. Moreover, Li et al. [5] concluded that all-cause and cardiac mortality rates were significantly lower in ESRD patients undergoing CABG than PCI, although early rates were higher in the same group. The authors observed no significant differences between CABG and PCI in CKD patients regarding late and cardiac mortality, but not early mortality, which was significantly higher with CKD patients undergoing CABG. Similarly, early mortality rate was significantly higher in ESRD patients undergoing CABG than others having PCI. However, late and cardiac mortality rates were significantly lower than the PCI group. This might suggest the better long-term efficacy of CABG than PCI.

The findings in our population are similar to the findings of the CKD population in the meta-analysis by Li et al. [5] since we found that CABG is inferior to PCI in early mortality with no advantage of either of them regarding late all-cause mortality. However, a definite conclusion cannot be drafted due to the various limitations to the current meta-analysis. The current literature is remarkably short on data regarding the appropriate revascularization strategy for KTRs. Our meta-analysis is the first of its kind to provide a comprehensive comparison about the superiority of either of PCI and CABG in KTRs. Comparably, more than ten meta-analyses were published comparing the same outcomes for ESRD and/or CKD patients [5]. Besides, no previous randomized controlled trials (RCTs) were found in the literature and relevant data could only obtained from four retrospective studies only. On the other hand, comparing revascularization strategies was reported among many studies for ESRD and/or CKD patients, although no RCTs were published in this context, as well. Revascularization outcomes are expected to be different among KTRs than ESRD and CKD patients since these patients usually have a higher risk for severe disease and various adverse health events [4]. This does not justify the current shortage of data regarding the best revascularization practice for KTRs.

To our knowledge, multiple pathologies can drive AKI in RTRs in particular infections and to a lesser extent, acute cardiovascular diseases [16]. In our study, we found that AKI prevalence was significantly higher in the CABG group rather than the PCI group. This observation can be explained by the long hospital stay in CABG patients which increase the susceptibility of acquiring hospital infections [9, 17]. Furthermore, the invasive technique of the CABG operation possesses a significant effect on the increase of the hospital stay in the KTRs rather than the PCI group.

There are some limitations to be considered before interpreting the current findings. First, the sample size of included studies and their included populations were small. Accordingly, a meta-analysis could not be conducted for some outcomes such as length of hospital stay and non-fatal graft failure, since they were reported by a single investigation. Second, all the analyzed data were obtained from retrospective studies which represented a major limitation since this type of data collection might jeopardize the quality of retrieved data. Thirdly, the designs of the included studies might be the best to compare these interventions due to significant factors, like population matching and heterogeneities regarding clinical and medical parameters, and follow-up periods. Accordingly, additional future investigations overcoming the current limitations are warranted for more proper validation of the current findings.

## Conclusion

It can be concluded that the risk of early mortality in KTRs is lower with PCI than CABG. However, it becomes comparable on a long-term basis. Furthermore, long-term survival probability is acceptable with both modalities, and choosing either of them over the other should be based on a wise clinical decision, other favorable outcomes, patient’s condition, and intended short or long-term outcomes. We hope our findings will help to establish relevant guidelines on the best revascularization practice for these patients. We also encourage future relevant investigations to be conducted for further validation of the current evidence.

## Supplementary Information

Below is the link to the electronic supplementary material.

Supplementary file1 (DOCX 19 KB)

## Data Availability

The data that supports the findings of this study are available from the corresponding author upon reasonable request.
