# Supplementary material for: Surgical versus interventional coronary revascularization in kidney transplant recipients: a systematic review and meta-analysis
Source: Int Urol Nephrol. 2023 Mar 12;55(10):2493–9. doi: 10.1007/s11255-023-03546-9 (PMC10499735; doi:10.1007/s11255-023-03546-9)
Supplement: Supplementary file 1 — Supplementary file1 (DOCX 19 KB) [file 11255_2023_3546_MOESM1_ESM.docx]

| Table S1: Search terms in each database | |
| --- | --- |
| Database | Search term |
| Pubmed | ("renal transplantation" OR "renal transplant") AND ("percutaneous coronary intervention" OR “PCI” OR "coronary catheterization" OR "coronary stenting") AND ("coronary artery bypass graft" OR "CABG") |
| ISI | ("renal transplantation" OR "renal transplant") AND ("percutaneous coronary intervention" OR “PCI” OR "coronary catheterization" OR "coronary stenting") AND ("coronary artery bypass graft" OR "CABG") |
| Scopus | ("renal transplantation" OR "renal transplant") AND ("percutaneous coronary intervention" OR “PCI” OR "coronary catheterization" OR "coronary stenting") AND ("coronary artery bypass graft" OR "CABG") |
| Scholar | With all of the words ("renal transplant" “coronary stenting”)  With at least one of the words ("coronary artery bypass graft" CABG "percutaneous coronary intervention" “PCI” “coronary catheterization”) |
| VHL | ("renal transplantation" OR "renal transplant") AND ("percutaneous coronary intervention" OR “PCI” OR "coronary catheterization" OR "coronary stenting") AND ("coronary artery bypass graft" OR "CABG") |

| Table S2: Quality rating of the included studies using National Institute of Health tool | | | | | | | | | | | | | | | |
| --- | --- | --- | --- | --- | --- | --- | --- | --- | --- | --- | --- | --- | --- | --- | --- |
| Reference ID | Q1 | Q2 | Q3 | Q4 | Q5 | Q6 | Q7 | Q8 | Q9 | Q10 | Q11 | Q12 | Q13 | Q14 | Total score |
| Charytan-2015-USA | 1 | 1 | 1 | 1 | 0 | 1 | 1 | 1 | 1 | 1 | 1 | 0 | 0 | 1 | 11 |
| Lang-2018-Germany | 1 | 1 | 1 | 1 | 0 | 1 | 1 | 1 | 1 | 1 | 1 | 0 | 0 | 1 | 10 |
| Taduru-2017-USA | 1 | 1 | 1 | 1 | 0 | 1 | 1 | 1 | 1 | 1 | 1 | 0 | 0 | 0 | 10 |
| Herzog-2004-USA | 1 | 1 | 1 | 1 | 0 | 1 | 1 | 1 | 1 | 1 | 1 | 0 | 0 | 0 | 10 |
| 1 yes, 0 = no, not applicable or not reported | | | | | | | | | | | | | | | |

1. Was the research question or objective in this paper clearly stated?
2. Was the study population clearly specified and defined?
3. Was the participation rate of eligible persons at least 50%?
4. Were all the subjects selected or recruited from the same or similar populations (including the same time period)? Were inclusion and exclusion criteria for being in the study prespecified and applied uniformly to all participants?
5. Was a sample size justification, power description, or variance and effect estimates provided?
6. For the analyses in this paper, were the exposure(s) of interest measured prior to the outcome(s) being measured?
7. Was the timeframe sufficient so that one could reasonably expect to see an association between exposure and outcome if it existed?
8. For exposures that can vary in amount or level, did the study examine different levels of the exposure as related to the outcome (e.g., categories of exposure, or exposure measured as continuous variable)?
9. Were the exposure measures (independent variables) clearly defined, valid, reliable, and implemented consistently across all study participants?
10. Was the exposure(s) assessed more than once over time?
11. Were the outcome measures (dependent variables) clearly defined, valid, reliable, and implemented consistently across all study participants?
12. Were the outcome assessors blinded to the exposure status of participants?
13. Was loss to follow-up after baseline 20% or less?
14. Were key potential confounding variables measured and adjusted statistically for their impact on the relationship between exposure(s) and outcome(s)?

- Good quality (12-14) points, fair quality (6-11) points and poor quality (0-5) points
